# Dose-Dependent Concentration and Penetration Depth of Cisplatin in Human Lung Tissue During Hyperthermic Exposure

**DOI:** 10.3390/jcm14030983

**Published:** 2025-02-04

**Authors:** Christopher Larisch, Till Markowiak, Martin Ignaz Schauer, Svitlana Golovchenko, Patrick J. Bednarski, Karolina Mueller, Christian Großer, Hans-Stefan Hofmann, Michael Ried

**Affiliations:** 1 Department of Thoracic Surgery, University Medical Center Regensburg, Franz-Josef-Strauss-Allee 11, 93053 Regensburg, Germanyhans-stefan.hofmann@ukr.de (H.-S.H.); michael.ried@ukr.de (M.R.); 2 Institute of Pharmacy, University of Greifswald, Friedrich-Ludwig-Jahnstrasse 17, 17489 Greifswald, Germany; bednarsk@uni-greifswald.de; 3 Center for Clinical Studies, University Medical Center Regensburg, Franz-Josef-Strauss-Allee 11, 93053 Regensburg, Germany; karolina.mueller@ukr.de; 4 Department of Thoracic Surgery, Hospital Barmherzige Brüder Regensburg, Prüfeninger Straße 86, 93049 Regensburg, Germany

**Keywords:** hyperthermic intrathoracic chemotherapy (HITOC), malignant pleural mesothelioma, thymoma, thoracic surgery, penetration depth

## Abstract

**Background**: Hyperthermic intrathoracic chemotherapy (HITOC) is an additive treatment option after surgical cytoreduction of pleural malignancies. Despite growing clinical experience and studies evaluating its feasibility, postoperative morbidity and mortality, as well as the effect on survival, there is still only little known about the local effects of HITOC on the lung parenchyma and tumour cells. The objective of this in vitro study was to evaluate the dose-dependent concentration and penetration depth of cisplatin in human lung tissue. **Methods**: In total, 40 patients were enrolled for elective lung resection, and wedge samples were taken to the laboratory. The visceral pleura was removed, and the decorticated lung tissue was incubated in cisplatin solutions of different concentrations (0.05, 0.075, and 0.1 mg/mL) at 42 °C over 60 min. Afterwards, platinum amounts in the lung tissue samples were measured using atomic absorption spectroscopy. **Results**: A strong decline of the cisplatin concentration was found until a depth of 3.5 mm, followed by a mild decline until a depth of 7.5 mm. In a depth of 0.5 mm, there was only a significant difference between 0.05 and 0.1 mg/mL (*p* = 0.03, Cohen’s d = 0.43). In a depth of 1.5 mm, there was an overall significant difference in cisplatin concentration dependent on dose (*p* = 0.027). In deeper tissue layers, no significant difference in cisplatin concentrations in the tissue was found. **Conclusions**: A dose-dependent increase of the cisplatin concentration was found for superficial tissue layers. This emphasises the relevance of sufficiently high intrathoracic concentrations of the chemotherapeutic agent. This study confirms that cisplatin penetrates lung tissue in therapeutically effective concentrations.

## 1. Introduction

A consistent challenge of thoracic surgery is to improve the treatment and long-term outcome of patients with malignant pleural mesothelioma and thymic tumours with pleural dissemination. Radical surgery was the choice of treatment for a long time, due to its diffuse growth. The goal was to achieve at least macroscopic complete resection.

In the last decade, surgical treatment of malignant pleural mesothelioma underwent a transition from radical resection such as extrapleural pneumonectomy (EPP; en-bloc removal of the parietal and visceral pleura, lung, pericardium, and diaphragm) towards lung tissue-conserving techniques such as pleurectomy/decortication (P/D) or extended (e)P/D (resection of pleura and, if necessary, pericardium, diaphragm, and only parts of the lung) [1,2,3]. The newly published MARS-2 trial even questioned the benefit of surgery itself in the multimodal treatment of malignant pleural mesothelioma, as the combination of surgery (P/D or eP/D) showed worse outcomes in comparison to chemotherapy alone concerning overall survival, adverse treatment events, and quality of life. Furthermore, surgical patients received less adjuvant chemotherapy or immunotherapy, probably due to deteriorated physiological condition postoperatively [4,5]. When comparing two even less invasive procedures (thoracoscopic partial pleurectomy versus talc pleurodesis, MesoVATS trial), again there was no significant difference in overall survival, but VATS caused more complications, longer hospitalisation, and higher costs [6]. At the moment, the MesoTRAP trial is recruiting patients comparing thoracoscopic partial pleurectomy versus indwelling pleural catheter in patients with trapped lung due to malignant pleural mesothelioma [7].

To be summarised, the role of radical surgery or even surgery itself is increasingly discussed by thoracic oncologists and surgeons, leading to the question of what surgery can offer in the future besides symptom control.

**Figure 1:**
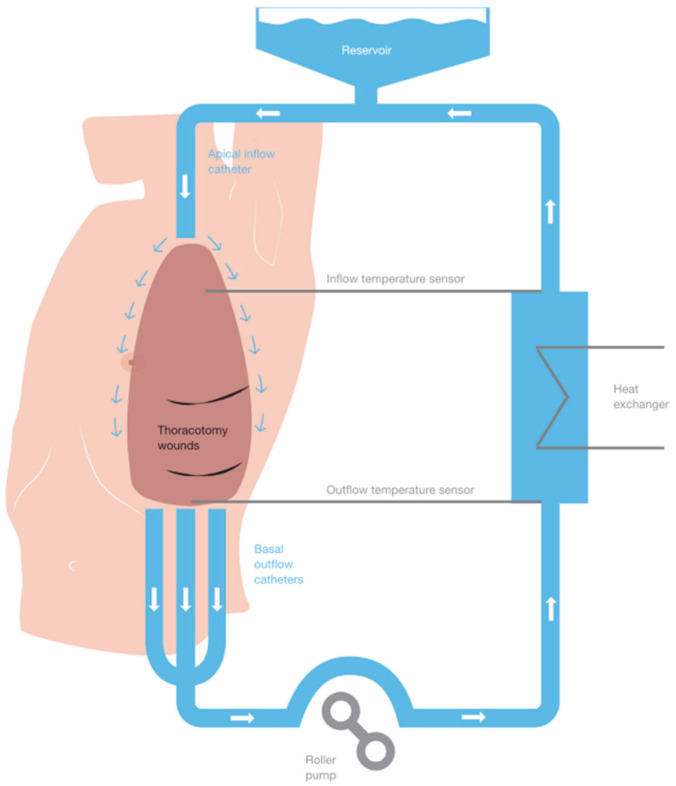
Schematic presentation of hyperthermic intrathoracic chemotherapy: one apical inflow tube and 3 basal outflow tubes, each with temperature sensors, connected to a perfusion system containing a reservoir (with isotonic solution and cytostatic drugs), a heat exchanger, and a roller pump [13].

Hyperthermic intrathoracic chemotherapy (HITOC) seems to be a promising answer. As already mentioned, due to its diffuse nature, tumour cells remain even after radical resection (such as EPP), leading to local and distant recurrences and thus poor prognosis [8]. This problem is countered by multimodal therapy, adding chemotherapy and radiotherapy to surgery. Concepts of additional intracavitary chemotherapy were developed to improve local tumour control by eradicating these remaining tumour cells after complete macroscopic resection. During HITOC, a hyperthermic (42 °C) cytostatic drug (cisplatin or a combination with doxorubicin) is applied intrapleurally over 60 min (Figure 1). The goal is to achieve a high local dose while avoiding side effects. There is increasing data on pharmacokinetics, clinical safety, and potential benefit in survival [9,10,11,12].

The concept of HITOC is also assessed in the modern setting of chemoimmunotherapy in clinical studies such as the NICITA trial [14].

Furthermore, HITOC is in the treatment of thymic malignancies with pleural dissemination. Due to low incidence, there are only small studies in the literature. It is not definitively clear if HITOC prolongs survival in this cohort, but HITOC possibly delays recurrence [15,16].

In the setting of malignant pleural effusion, HITOC is only used in selected cases. As malignant pleural effusion is considered an advanced disease and prone to or simultaneous with further distant metastases and HITOC being a regional treatment option, there are only a few studies in the literature. In a systematic review, one group assessed the efficacy in patients with non-small cell lung cancer (NSCLC) with N0-1 disease and malignant pleural effusion (M1a), corresponding to a tumour stage of UICC IVA. They only identified 21 patients with sufficient data; the median survival was 18 months, and the treatment-related mortality was 0% [17]. Another feasibility study examined EPP and HITOC (using an extreme cisplatin dose of 200 mg/m^2^ BSA) in patients with T4 N0-1 NSCLC with a mean survival of 19 months [18]. A meta-analysis done by Zhou et al. [19] favoured the combination of cytoreductive surgery and HITOC over surgery alone in lung cancer, thymic malignancies, breast, and ovarian cancer concerning overall survival and tumour-free survival [19]. The potential benefit of radical or cytoreductive surgery combined with HITOC in lung cancer with pleural spread as well as extra-thoracic malignancies with manageable disease (within an oligometastatic concept) remains to be examined more rigorously in the future.

However, there is still only little knowledge concerning the local effects of the administered chemotherapeutic drugs, with many questions still to be answered, for example: What is the optimal combination and dose of cytostatic drugs? What is the optimal temperature and duration of the perfusion? Does perfusion pressure play a role in the penetration depth of cisplatin? Do HITOC conditions overcome natural resistance patterns of malignant pleural mesothelioma [20,21,22].

Cisplatin is used as a standard in intrathoracic chemotherapy, but doses for intrathoracic application in HITOC vary. For these reasons, important aspects of the intrathoracic effects of different doses of cisplatin, especially on the depth of penetration into the lung tissue, could still not be answered. Earlier analyses by our research groups were already able to demonstrate a significant dependence of the penetration depth of cisplatin in particular on the decortication of the lung tissue with a simultaneous significant lack of influence of hyperthermia, potentially due to a small study population [23,24]. Unfortunately, there are no conclusive data from clinical in vivo examinations after P/D and HITOC. This is also since assessing local effects of intrathoracic hyperthermic perfusion is difficult as the perfusion is performed on the closed chest without easy access to perform biopsies and with the danger of exposing medical staff to high doses of cytostatic drugs [25,26,27].

We established an in vitro model for experimental analysis of the HITOC procedure to gain knowledge of local effects of cisplatin on healthy human lung tissue. A first explorative in vitro study of our working group showed that the penetration depth of cisplatin in human lung tissue with intact visceral pleura was approximately 3–4 mm [24].

In the next step, we took a step closer to clinical settings and compared cisplatin concentration and penetration depth in dependence on decortication and various temperatures [23]. Here we found that decortication resulted in a significant difference in cisplatin concentration in the tissue, while there was no significant difference dependent on temperature. Another ex vivo study emphasises the importance of dose, combination of cytostatic drugs, and temperature on the survival of mesothelioma cell lines, whereas the duration of treatment (exposure to cytostatic drugs) was not decisive [28]. Further data are inconsistent in whether MPM cells are sensitive or rather resistant to commonly applied concentrations of cisplatin [21,29].

The objective of this in vitro study was to evaluate the impact of various cisplatin doses in the perfusate on cisplatin concentration and penetration depth in decorticated human lung tissue.

## 2. Materials and Methods

### 2.1. Study Design

This experimental study was approved by the local Ethical Committee of the University of Regensburg (reference number: 19-1379-101) in April 2019. The trial was conducted in accordance with the Declaration of Helsinki (as revised in 2013). All patients signed an informed consent before participating in the study. Criteria for inclusion were an age older than 18 years, elective lung resection, and no obtained neoadjuvant therapy. Patients’ data were pseudonymised.

### 2.2. Experimental Setting

The following methodical approach can also be read in a previous paper of our working group [23]. We obtained human lung tissue samples by performing a wedge resection of healthy tissue on the resected lobes under ex vivo conditions in the operating theatre. The samples were transported into the laboratory without embedding in any fluid for further processing within 3 h. A decortication of the visceral pleura was performed, and the lung tissue samples were incubated with solutions of various concentrations of cisplatin (0.05, 0.075, and 0.1 mg/mL) at 42 °C for 60 min.

A cisplatin concentration of 0.05 mg/mL represents a moderate cisplatin dose of 100 mg/m^2^ BSA (body surface area). The concentration of 0.075 mg/mL translates into an increased dose of 175 mg/m^2^ BSA, which is used in our hospital. Finally, the concentration of 0.1 mg/mL would be a dose of 200 mg/mL, which we examined only for comparative research, as it is not used clinically because of the increased risk for renal damage. The tissue samples were frozen in liquid nitrogen, and subsequent slices of 50 µm were prepared with a cryomicrotome (Leica CM1900; Leica Biosystems, Wetzlar, Germany). Then ten slices each were put in a 1.5 mL Eppendorf cup (representing a tissue diameter or depth of 0.5 mm) and incubated in a solution of 0.9% NaCl and 65% nitric acid. Afterwards, homogenisation was performed (MP Biomedicals FastPrep-24; BIOZOL, Heidelberg, Germany). We incubated the cups in a water bath at 80 °C for 24 h or until there were clear solutions. 30 µL of the content from each cup were diluted with 970 µL of water (dilution of 1:33). Then the mass concentration of platinum was measured with graphite furnace atomic absorption spectroscopy (AAS) at the University of Greifswald. Every sample was analysed in triplicate with a relative standard deviation of 5% or less. For each measurement series, a calibration with eight standards between 14.5 and 162 µg/L of platinum was performed by using a quadratic function. Correlation coefficients (r) for the calibration curves were >0.997. Given the principle of graphite furnace AAS, we detected platinum instead of cisplatin itself. Thus, for better comparability with the common literature, the measured platinum concentrations were converted into cisplatin concentrations by using the molar equation. Provided that platinum is not or only in negligible quantity in the human body, we assume the measured platinum dose is equal or at least near to the exposed cisplatin dose. In a last step, the data of cisplatin concentration were transformed from µg/mL (mass of cisplatin per volume of solution) into µg/mg (mass of cisplatin per mass of lung tissue). In addition, the current data were compared with previous data of our study group.

We compared the cisplatin concentrations measured in the superficial tissue samples in dependence on the cisplatin concentrations in the perfusate (0.05 vs. 0.075 vs. 0.1 mg/mL). Thereby we measured the cisplatin concentration in every “unit of penetration depth” (0.5, 1.5, 2.5 mm, etc.).

### 2.3. Statistical Analysis

Data collection was conducted using Microsoft Office 365 Excel, and statistical analysis was performed via IBM SPSS Statistics 29. The samples were characterised per lab parameters (absolute frequency [n], median [med], interquartile range [IQR], minimum, maximum). Due to the small sample size and partially not normally distributed data, Kruskal–Wallis tests were used to compare cisplatin concentrations in the lung tissue between three cisplatin doses (0.05 mg/mL, 0.075 mg/mL, and 0.1 mg/mL) at each depth layer. The two-sided level of significance was set at *p* ≤ 0.05. Adjustment for multiple testing was not performed as the analysis was of an exploratory manner.

## 3. Results

**Figure 2:**
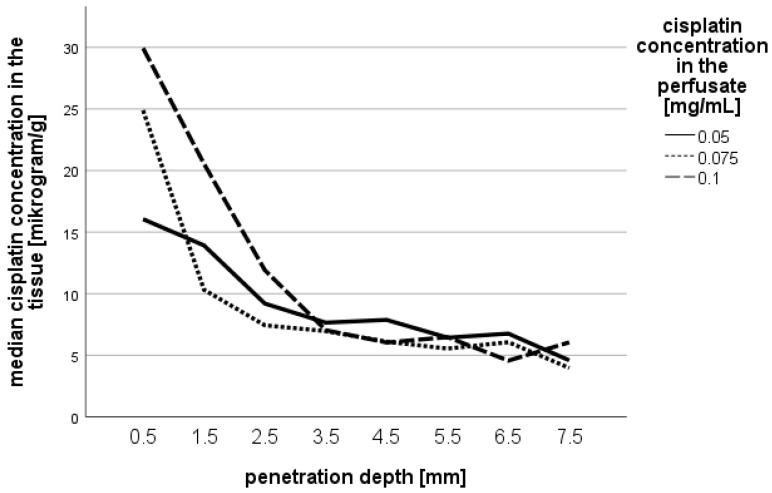
Median cisplatin concentration in the lung tissue proportional to penetration depth depending on the cisplatin dose in the perfusate.

In total, 40 cases with wedge resections of human lobectomy specimens were included. Experimental investigations were conducted with different doses of cisplatin (n = 13 at 0.05 mg/mL; n = 15 at 0.075 mg/mL; n = 12 at 0.1 mg/mL). All concentrations of cisplatin related to the tissue slices are presented in Table 1 and Figure 2. It is to be mentioned that most samples existed for the superficial layers, whereas in the deeper tissue there were fewer samples.

In general, the highest cisplatin concentration was found in the first (most superficial) layer (depth of 0.5 mm). A strong decline of the cisplatin concentration was found till a depth of 3.5 mm, followed by a further mild decline till a depth of 7.5 mm. The overall maximum penetration depth was 7.5 mm due to limitations of our method.

**Figure 3:**
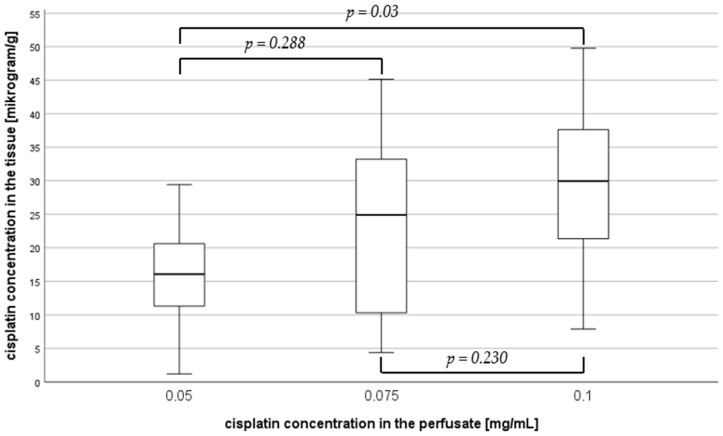
Median cisplatin concentration in a penetration depth of 0.5 mm depending on the cisplatin dose in the perfusate.

In the depth of 0.5 mm (Figure 3), the median cisplatin concentration was 16.05 µg/g (IQR 10.97–22.72) at a cisplatin dose of 0.05 mg/mL, 24.89 µg/g (IQR 10.28–40.34) at a dose of 0.075 mg/mL, and 29.94 µg/g (IQR 15.73–38.96) at a dose of 0.1 mg/mL. This corresponds to an increase in cisplatin concentration of over 87%. There was a significant difference in the subgroup comparison between 0.05 and 0.1 mg/mL (*p* = 0.03) with a medium effect size (Cohen’s d = 0.43).

**Figure 4:**
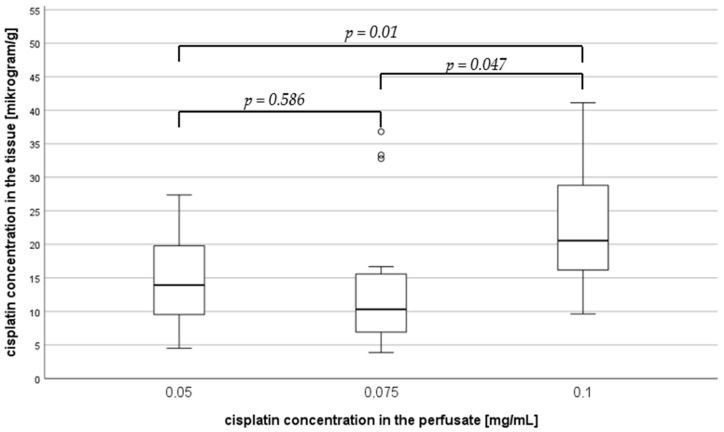
Median cisplatin concentration in a penetration depth of 1.5 mm depending on the cisplatin dose in the perfusate.

In the depth of 1.5 mm (Figure 4), the median cisplatin concentration was 13.92 µg/g (IQR 9.51–20.42) at a cisplatin dose of 0.05 mg/mL, 10.32 µg/g (IQR 6.80–16.69) at a dose of 0.075 mg/mL, and 20.56 µg/g (IQR 15.97–30.33) at a dose of 0.1 mg/mL. This corresponds to an increase in cisplatin concentration of over 48%. There was an overall significant difference in cisplatin concentration dependent on dose (*p* = 0.027). Subgroup analysis showed significant differences between the doses of 0.05 and 0.01 mg/mL (*p* = 0.01) as well as between 0.075 and 0.1 (*p* = 0.047), both with a medium effect size (Cohen’s of 0.3 and 0.4, respectively).

For deeper tissue layers, there were no detectable significant differences. At a penetration depth of 2.5 mm, there was only an increase of cisplatin of 29% by higher dosage; at a depth of 5.5 mm, the concentrations were equal.

## 4. Discussion

The HITOC procedure contains multiple parameters the surgeon can vary, including (the extent of) pleurectomy, temperature of the perfusate, dosage of cisplatin, combination of multiple chemotherapeutic drugs, and duration of chemotherapy perfusion. Each issue is discussed in the following.

In a previous in vitro study of our working group, we explored the effect of pleurectomy and hyperthermia. We found that the overall maximum penetration depth was 7.5 mm due to limitations of our methods. The cisplatin concentration decreased with increasing penetration depth (*p* < 0.001). Pleurectomy at 42 °C significantly increased the cisplatin concentration in comparison to tissue samples with intact pleura (*p* = 0.005) by a constant difference of 1.34 µg/mL. This corresponds to an increase in cisplatin concentration of over 57% in the most superficial tissue layers. In contrast, an increase in temperature showed no significant effect on the cisplatin concentration in decorticated tissue samples (*p* = 0.985), potentially due to the small size of our study cohort. Similarly, the functional maximum penetration depth (defined as the very penetration depth with a cisplatin concentration of at least 1 µg/mL [28]) was not influenced by temperature (*p* = 0.243) but by pleurectomy (*p* < 0.001) [23]. However, this does not mean that hyperthermia does not play an important role in HITOC, as studies have shown that higher temperatures in combination with cytostatic drugs resulted in lower survival rates of mesothelioma cells in vitro. This effect, however, decreased with increasing cytostatic efficacy and accounted for only 0 to 28% of the reduction of viable cancer cells [28]. Translating these in vitro data onto our results, the survival rate of mesothelioma cells would be approximately 10–40% in superficial depths to 1.5 mm as well as 40–70% and worse in tissue levels of 5.5 mm and deeper.

It is common practice to perform the HITOC over 60 min. In the German PLEURATUMOR database, 89.7% of all HITOCS were performed over 60 min. Only 6.1% lasted for 90 min and 0.6% took 120 min (unpublished data).

In contrast, doses of cisplatin used for intrathoracic perfusion vary more broadly. In Regensburg, we increased the cisplatin dose from initially 100 mg/m^2^ BSA (body surface area) (comprising an in vitro dose of approximately 0.05 mg/mL) to 175 mg/m^2^ BSA (or 0.075 mg/mL in vitro) without a relevant worsening of clinical side effects (especially acute renal failure) using measures for renal protection [30]. In this experimental study, we additionally added a cisplatin dose of 0.1 mg/mL (or approximately 200 mg/m^2^ BSA). We found that higher doses of cisplatin in the perfusate resulted in higher cisplatin concentrations in the tissue, but only in the superficial layers. As mentioned in a previous paper, the limitation of the method does not allow for any statement about the absolute penetration depth of cisplatin, defined as that penetration depth where cisplatin concentration reaches zero [23,24].

Due to the German PLEURATUMOR database a single cytostatic drug (cisplatin) was applied in 55.4% of patients; in 43.4% a combination was used (cisplatin and doxorubicin), in 62 patients a triple combination was used (unpublished data). From in vitro studies, we know that the combination significantly enhances the reduction of viable tumour cells. The combination of cisplatin and gemcitabine reduced the survival rate from 0 to 9.6%; the combination of cisplatin and pemetrexed from 0 to 4.5%. In comparison, the incubation in cisplatin only reduced the survival rate from 13.6 to 26%. Sadly, the most common combination of cisplatin with doxorubicin was not examined [28].

As explained above, it is difficult to obtain in vivo samples after HITOC from the patient. Nevertheless, we found two studies in the literature in which this was achieved. In one study by Ratto et al. they obtained lung biopsies after hyperthermic perfusion (41.5 °C over 60 min) with cisplatin (100 mg/m^2^ BSA) and measured a concentration of 5.25 µg platinum/g lung tissue [31]. The exact depth of the biopsy was not mentioned. This would be roughly 8 µg cisplatin/g lung tissue. In contrast, we measured a double cisplatin concentration at a dose of only 0.05 mg/mL and a 3.6-fold increase at a dose of 0.1 mg/mL in the superficial tissue layer of 0.5 mm (Table 1). Even in the deep tissue layer of 5.5 mm, we measured a median cisplatin concentration of 6.26 µg/g, averaged over all cisplatin doses used in this analysis. With almost the same parameters used, you can conclude that Ratto et al. took deeper lung biopsies, which explains those rather low cisplatin concentrations in their tissue samples. Thus, our study could be considered a more detailed analysis of cisplatin concentration in superficial tissue layers. Another study was performed by Opitz et al., who investigated the effects of intrathoracic platin-fibrin layers in the local therapy of pleural mesothelioma [32]. Unfortunately, a direct comparison with our results is difficult due to two facts. First, the surgeons used a special cisplatin-fibrin solution sprayed onto the lung surface without using hyperthermia, which may have benefits because the cytostatic drug impacts remaining tumour cells for a prolonged time. In addition, no data about the depth of biopsies was given. They measured cisplatin doses from approximately 12 up to 72 µg/g, which is far higher than the concentrations we found in the lung tissue. In our opinion, this could be due to the longer duration of exposure (90 vs. 60 min) as well as the special cisplatin-loaded fibrin with remaining gel on the lung surface at the time of the biopsy, thus creating false high results. Comparisons to in vitro cell culture studies are quite difficult because we measured cisplatin concentration per weight of tissue, whereas in cell culture you assess effects using cisplatin concentrations per volume of solution [28].

The results of this study comprise some limitations. First, although this was an in vitro study within a highly controlled setting, there was quite a variability in the quality and size of tissue samples. Second, the penetration depth and concentration could be different in vivo, although we sought fast processing of the tissue sample once they were resected. This could be, for example, due to beginning necrosis of the tissue sample, absent flow and pressure in the solution such as in HITOC with its perfusion system, and absent intraparenchymal blood flow such as in vivo.

Statements on the local effectiveness of intrathoracic cisplatin on tumour cells in superficial layers of lung tissue can hardly be made on the basis of our experimental setting and, despite the difficulties mentioned above, require further experimental and clinical studies. Currently, our working group is building a perfusion system to incubate both wedge lung samples from patients and cell cultures of malignant mesothelioma and thymoma to measure cell survival under HITOC-like conditions. We hope to answer the question of whether perfusion pressure plays a role in the penetration depth of cisplatin in human lung tissue. Beyond that, we are performing cell culture experiments to assess the effectiveness and resistance patterns of cisplatin and doxorubicin (the most commonly used combination in HITOC). These are the next steps to emulate HITOC ex vivo and to measure its local effects.

## 5. Conclusions

A dose-dependent increase of the cisplatin concentration was found for superficial tissue layers. This emphasises the relevance of sufficiently high intrathoracic concentrations of the chemotherapeutic agent. This study confirms that cisplatin penetrates lung tissue in therapeutically effective concentrations.

## Figures and Tables

**Table 1:** Penetration depth and cisplatin concentration in human lung tissue depending on the cisplatin dose in the perfusate. CT: cisplatin concentration in the tissue [µg/g], CP: cisplatin concentration in the perfusate [mg/mL], mm: millimetre, IQR: interquartile range.

CT at	CP = 0.05			CP = 0.075			CP = 0.1		
	n	Median	IQR	n	Median	IQR	n	Median	IQR
0.5 mm	13	16.05	10.97–22.72	15	24.89	10.28–40.34	12	29.94	15.73–38.96
1.5 mm	13	13.92	9.51–20.42	15	10.32	6.80–16.69	12	20.56	15.97–30.33
2.5 mm	12	9.20	5.69–13.23	15	7.43	5.85–15.72	12	11.91	4.25–18.12
3.5 mm	13	7.65	4.10–10.57	15	6.96	4.85–9.64	12	7.04	6.00–11.14
4.5 mm	11	7.87	3.96–10.03	14	6.10	4.95–8.66	10	6.04	4.84–9.35
5.5 mm	5	6.43	4.81–9.45	12	5.54	4.15–8.93	7	6.47	4.44–11.62
6.5 mm	2	6.76	6.14–7.37	8	6.06	2.61–7.94	5	4.56	2.11–11.32
7.5 mm	2	4.59	3.51–5.66	1	3.97	---	1	6.05	---

## Data Availability

Data are available on request due to privacy and ethical restrictions.
